# From the gut to the brain, mechanisms and clinical applications of γ-aminobutyric acid (GABA) on the treatment of anxiety and insomnia

**DOI:** 10.3389/fnins.2025.1570173

**Published:** 2025-05-07

**Authors:** Chengji Jiang, You Chen, Tao Sun

**Affiliations:** ^1^Center for Precision Medicine, School of Medicine and Biomedical Sciences, Huaqiao University, Xiamen, China; ^2^GeneYoung Biopharmaceuticals, Shenzhen, China

**Keywords:** GABA, anxiety, insomnia, gut microbiota, probiotics, genetic engineered bacteria

## Abstract

Anxiety and insomnia are prevalent global mood disorders, and affect approximately 4 and 10 out of every 100 individuals, respectively. Common abnormal brain activity and altered neural circuitries are detected in patients with anxiety disorders and insomnia, suggesting overlapping pathogenesis in these two disorders. Promisingly, GABA from dietary supplements and GABA produced by gut microbiota have shown significant treatment effects in anxiety and insomnia. This review summarizes neurological mechanisms causing anxiety and insomnia, reveals cellular pathways transferring GABA from the gut to the brain, and delivers the therapeutic potential of gut derived GABA for anxiety and insomnia. Moreover, this review proposes emerging therapeutic strategies utilizing engineered GABA-producing bacteria to target anxiety and insomnia, and highlights the potential of live biotherapeutics as novel interventions for mood disorders.

## 1 Introduction

The global prevalence of anxiety disorder is increasing, particularly in cities with greater levels of stress and fast-paced lifestyles. In 2024, nearly 275 million people are suffering from anxiety disorders, accounting for approximately 3.5% of the global population (Calm, 2024). Moreover, about 10% of adults are affected by insomnia disorders, and an additional 20% experiences occasional insomnia symptoms worldwide (Morin and Jarrin, 2022). Anxiety disorders and sleep disturbances exhibit a bidirectional relationship, where anxiety-induced hyperarousal disrupts sleep architecture, and sleep deprivation amplifies emotional vulnerability (Bragantini et al., 2019; Pillai et al., 2014; Sadeh et al., 2004). Specifically, sleep deprivation increases cortisol levels, amplifies emotional procession of the limbic system and the salience network involved in cognitive control (dorsal anterior cingulate cortex and anterior insula), and creates a vicious cycle of hyperarousal and anxious apprehension (Carlisi et al., 2017; Palmer and Alfano, 2020; Yoo et al., 2007). Conversely, anxiety disorders disrupt sleep architecture by reducing slow-wave sleep and increase nocturnal awakenings through noradrenergic hyperactivity in the locus coeruleus (Chellappa and Aeschbach, 2022; Gong et al., 2021).

Pathogenic studies have shown that anxiety and insomnia share similar biological mechanisms (Morris et al., 2020; Van Someren, 2021). The locus coeruleus (LC) can activate the amygdala by releasing norepinephrine (NE), thereby inducing anxiety and triggering the body’s anxious response (Suárez-Pereira et al., 2022). The locus coeruleus also acts as the control center for wakefulness. Its specialized nerve cells release norepinephrine when one is awake, like an internal “alarm system.” During deep and slow-wave sleep, these cells quiet down significantly, and fall silent completely during rapid eye movement (REM) sleep, the stage mostly associated with vivid dreaming (Verret et al., 2006). Thus, the LC serves as a pivotal point where the two disorders converge.

Another potential pivotal point is the hypothalamic–pituitary–adrenal (HPA) axis. Anxiety and insomnia patients commonly exhibit activation of the HPA axis, characterized by elevated hormone levels such as cortisol (Juruena et al., 2020; Zhang et al., 2014). Elevated cortisol levels enhance the amygdala’s sensitivity to threat signals (Roberts et al., 2022). Prolonged high cortisol exposure keeps it in “hyper-vigilance,” causing continuous anxiety even without real threats. Moreover, high cortisol can activate the amygdala, and reduce slow-wave sleep (Buckley and Schatzberg, 2005; Wong et al., 2000). Additionally, increased cortisol damages prefrontal cortex neurons and weakens its inhibitory function on the amygdala, impairing one’s emotion regulation ability (Ren et al., 2022).

Currently, besides behavioral interventions such as mental therapy and exercise, medication remains an effective treatment for anxiety and insomnia (Kandola and Stubbs, 2020; Riedel et al., 2024; Shaha, 2023; Vu and Conant-Norville, 2021). Many medications have been developed, with several already commercialized and available on the market. For example, classic medications such as barbiturates and benzodiazepines are known to possess sedative and hypnotic effects (Bormann, 1988; Bowery et al., 1979; Sigel and Ernst, 2018). However, the current medications for anxiety and insomnia often cause various side effects. For instance, benzodiazepines can cause sedation, slowed reaction time, and impaired memory and psychomotor function (Greenblatt et al., 2020). Therefore, the development of a more natural and less harmful medication holds significant promise for the treatment of anxiety and insomnia.

As a natural neurotransmitter, γ-aminobutyric acid (GABA) is promoted for its benefits in addressing anxiety and insomnia (Hepsomali et al., 2020; Ngo and Vo, 2019). The efficacy of orally administered GABA is supported by positive consumer reviews and clinical experiments, suggesting its promising therapeutic effects on anxiety and insomnia symptoms (Ngo and Vo, 2019). Dietary sources that are rich in GABA and GABA-producing probiotics also display significant therapeutic effects on anxiety and insomnia (Liwinski et al., 2023; Strandwitz, 2018).

Because the association of gut microbiota in human health and diseases is well recognized, it is timely to better understand how gut derived GABA may function in humans. Moreover, in this review, orally administered GABA, GABA from GABA-rich food, and GABA from GABA-producing probiotics are collectively referred to as “gut derived GABA.” This review explores production, absorption and transmission of gut derived GABA, clarifies its potential mechanisms in alleviating anxiety and insomnia, and discusses future applications for optimized production and clinical use.

## 2 Anxiety and insomnia

### 2.1 Anxiety

Anxiety disorder represents a prevalent category of mood disorders (Byrd and Brook, 2014). While acute anxiety involves a sudden onset of intense fearful experiences, chronic anxiety is the most common presentation of anxiety disorders, with symptoms including persistent and unwarranted worries, fatigue, insomnia and profound distress (Nutt, 2005; Robinson et al., 2019). A variety of risk factors, including family issues, environmental exposures, and perceived threats, can contribute to the development of anxiety (Warner and Strawn, 2023).

The neural circuitry associated with anxiety has been studied. The amygdala plays a crucial role in emotion generation, recognition and regulation, as well as in controlling learning and memory, and fear response through receiving and responding information from the locus coeruleus (LC) and other sensory inputs (Krettek and Price, 1978; Suárez-Pereira et al., 2022). For transient panic, the basolateral amygdala (BLA) integrates sensory information from the environment and activates the central amygdala (CE). Subsequently, the central nucleus of the amygdala (CeA) triggers defensive responses by projecting to brain regions such as the ventral striatum, hippocampus, lateral hypothalamus (LH) and surrounding areas (LeDoux, 2000). For persistent anxiety, the bed nucleus of the stria terminalis (BNST), which is part of an extended amygdala, is more directly involved and closely coupled with the CE (Zhu et al., 2024).

Under high-pressure conditions, the LC enhances amygdala function while simultaneously attenuates the prefrontal cortex (PFC) function to facilitate fear learning. Conversely, under low-pressure conditions, the LC enhances PFC function, thereby increasing the inhibitory effect of the medial prefrontal cortex (mPFC) on the amygdala from top to bottom, and subsequently promoting fear extinction (Giustino and Maren, 2018).

Moreover, the hippocampus receives signals from the BLA and stores fear and long-term anxiety signals (Hajisoltani and Meftahi, 2024). These memories persist for a period and can be reactivated by a similar context, even if the individual is no longer directly in that situation (Chaaya et al., 2018). In addition to anxiety signals from the locus coeruleus, sensory information from visceral changes, such as heart rate, gastrointestinal responses and blood pressure, can be transmitted to the mPFC via the anterior insula, thereby contributing to the maintenance of anxiety (Vertes, 2004). Disrupted limbic-hypothalamic–pituitary–adrenal axis (LHPA), commonly referred to as the HPA axis, is also involved in anxiety disorders. Patients with anxiety exhibit elevated levels of plasma cortisol, which is released from the adrenal gland, potentially intensifying feelings of fatigue due to the acceleration of glycogen metabolism (Juruena et al., 2020).

### 2.2 Insomnia

Insomnia is the most prevalent sleep disorder in industrialized countries, characterized by symptoms such as dissatisfaction with sleep quality or duration, difficulty falling asleep at bedtime, waking up in the middle of the night or too early in the morning (Morin and Benca, 2012). Although symptoms of insomnia primarily occur at night, many individuals also experience daytime cognitive impairments, such as difficulties with attention, concentration and memory, as along with mood disturbances like irritability and dysphoria (Buysse et al., 2007). Even though a significant number of adults are suffering from severe insomnia, less than 15% of those receive proper treatment (Bhat et al., 2008).

Dysfunction of the wakefulness and sleep neural systems is the major cause of insomnia (Riemann et al., 2010). The wakefulness regulatory system comprises two pathways (Saper et al., 2005). One pathway involves NA neurons in the LC, serotonin (5-HT) neurons in the raphe nuclei, histaminergic (His) neurons in the tuberomammillary nucleus (TMN) and dopaminergic (DA) neurons in the ventral periaqueductal gray (vPAG) (John et al., 2004; Ko et al., 2003; Verret et al., 2006). This pathway receives signals from neurons in the LH, including orexin (ORX) and melanin-concentrating hormone (MCH), and acetylcholine (ACh) and GABA from neurons in the basal forebrain (BF), and leads to cortical activation and wakefulness. The second ascending arousal pathway includes cholinergic neurons in the pedunculopontine tegmental nucleus (PPT) and laterodorsal tegmental nucleus (LDT). Their activation stimulates thalamic relay neurons, resulting in cortical activation and maintenance of conscious wakefulness (Morin et al., 2015).

In addition, the sleep-promoting system includes the hypothalamic ventrolateral preoptic area, basal ganglia, cerebral cortex, limbic system, BF, and the brainstem and thalamus (Saper et al., 2005). Activation of GABAergic neurons in the ventrolateral preoptic nucleus (VLPO) and inhibition of NA activity mediated sleep are the core sleep-promoting cluster (Dubourget et al., 2017). Moreover, VLPO GABAergic neurons can inhibit the activation of wakefulness systems, including the LDT, dorsal raphe nucleus (DRN), and locus coeruleus (LC), causing a transition to sleep (Morin et al., 2015).

Sleep–wake homeostasis is maintained by the “Flip-Flop Switch” model, in which transition between sleep and arousal depends on interactions among the VLPO, monoaminergic cell groups (nuclei) and ORX neurons (Morin et al., 2015; Saper et al., 2005). Dysregulation of the arousal system, characterized by heightened physiological arousal during both sleep and wakefulness, results in a state known as hyperarousal, which is a significant contributor to insomnia (Riemann et al., 2010).

## 3 An association of GABA with anxiety and insomnia

Insomnia is considered to exacerbate anxiety, since inadequate sleep can increase negative emotions, diminish positive emotions, and alter adolescents’ comprehension, expression and modulation of emotions (Blake et al., 2018; Palmer and Alfano, 2017). Conversely, anxiety can contribute to the onset of insomnia, and stress exposure disrupts sleep, leading to difficulties in both falling asleep and staying asleep (Kalmbach et al., 2018).

It appears that both anxiety disorder and insomnia are associated with decreased levels of GABA. Significantly reduced GABA levels have been observed in brains of individuals with anxiety disorders, particularly in the thalamus and the amygdala, as detected by Magnetic Resonance Spectroscopy (MRS) analysis (Babaev et al., 2018; Streeter et al., 2010). GABA levels in plasma of patients with anxiety have shown notable decreases (Lydiard, 2003; Shutta et al., 2021). Moreover, reduced expression levels of GABA_A_ receptor α1 and α2 subunits have also been detected in the serum of insomnia patients (Xiang et al., 2023). On the other hand, the GABA_A_ receptor agonist, such as benzodiazepine, can effectively alleviate symptoms of anxiety and treat insomnia, and the antagonist of GABA_B_ and GABA_C_ receptors, like CGP-36742, can reduce a patient’s slow-wave sleep (Deschaux et al., 2006; Nutt, 2005; Sternbach, 1978).

## 4 Synthesis and functions of GABA

**Figure 1:**
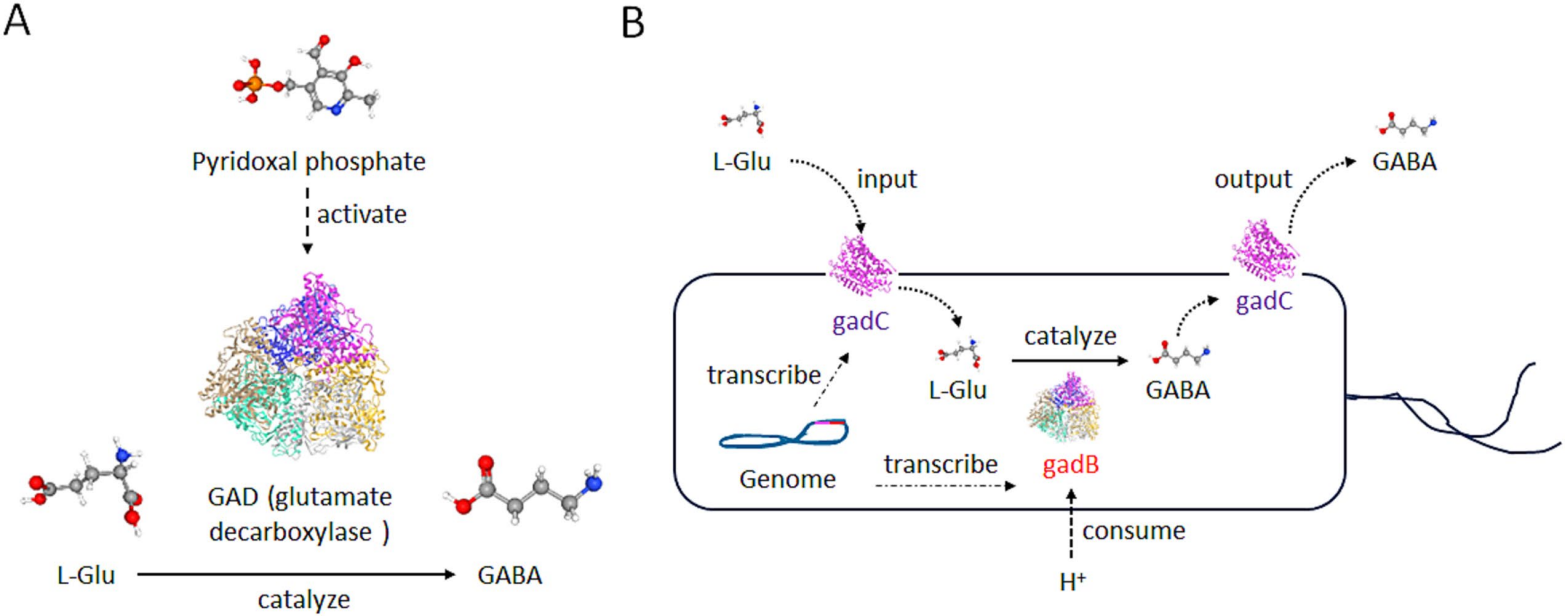
Structure and production of GABA. **(A)** The production diagram of GABA. With the assistance of pyridoxal phosphate, the glutamate decarboxylase (GAD) enzyme is transformed into an active structure and then converts L-Glu into GABA. L-Glu, GABA, and Pyridoxal phosphate are illustrated by PubChem. White ball: the hydrogen atom; red ball: the oxygen atom; gray ball: the carbon atom; blue ball: the nitrogen atom; yellow ball: the phosphorus atom. **(B)** The diagram of GABA’s production by gut microbiota. The genome of many bacteria carries the gadB and gadC genes. The gadB is a type of GAD in **(A)**, and functions in the same manner as GAD. GABA produced by gadB can be transported extracellularly through gadC. Meanwhile, the raw material L-Glu can also enter the cell through gadC, and this process can consume H^+^ ions outside the environment.

GABA is a prominent neurotransmitter within the brain, and also functions as a regulatory hormone in peripheral organs, which holds significant importance for the human body (Jin and Korol, 2023; Zhang et al., 2021). GABA, also known as 4-aminobutyric acid, is a compound represented by the chemical formula C₄H₉NO₂ (Figure 1A). It is an amino acid that is widely distributed among vertebrates, plants, and microorganisms (Sarasa et al., 2020). In living organisms, GABA is primarily synthesized by glutamate decarboxylase (GAD), an enzyme that uses glutamate (L-Glu) as the raw material in the presence of coenzyme pyridoxal phosphate and protons (Figure 1A) (Soghomonian and Martin, 1998).

**Figure 2:**
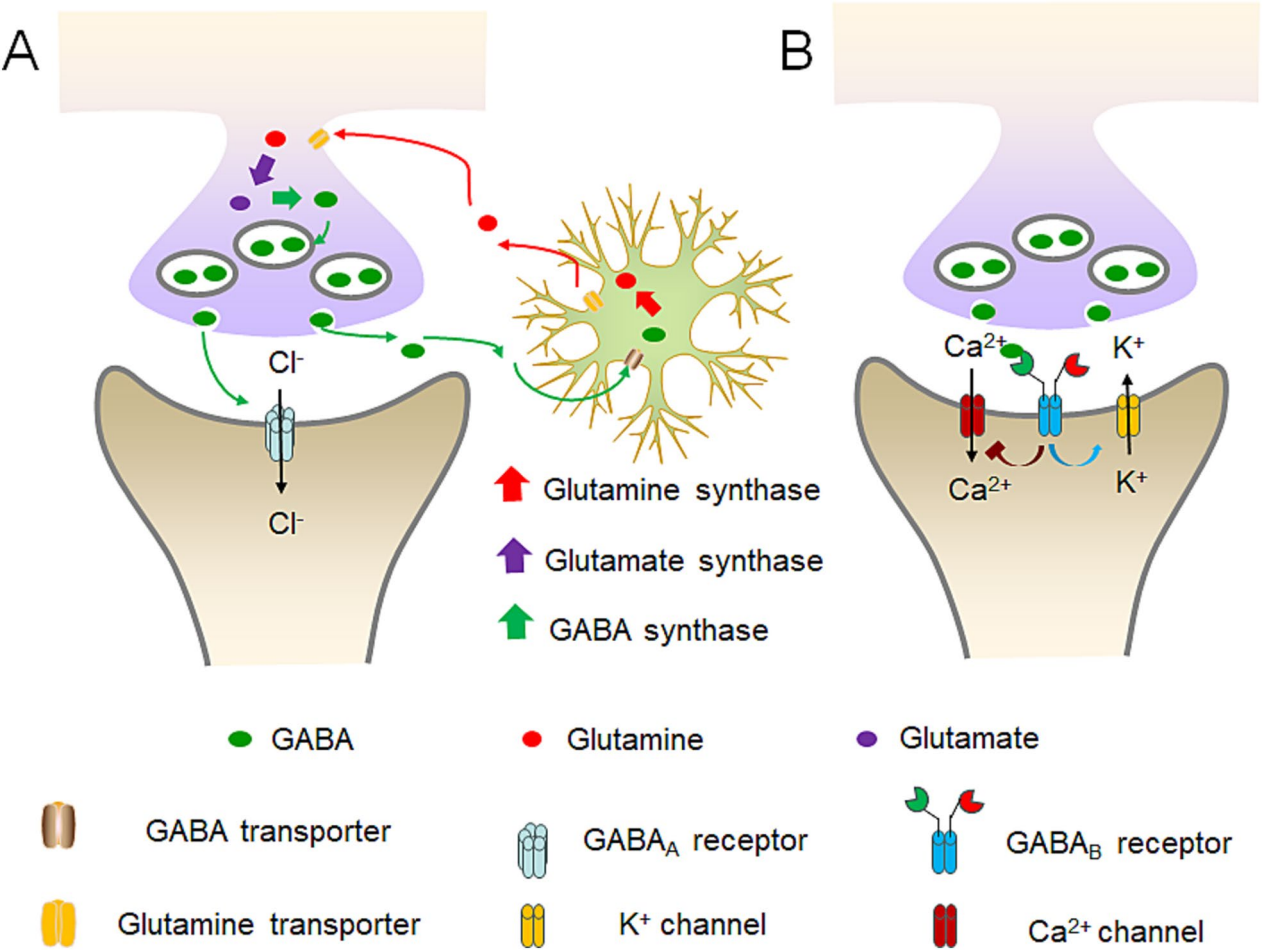
Diagram of GABA receptors’ workflow and GABA’s conversion balance. **(A)** GABA_A_ receptors’ workflow and the balance among GABA, glutamine and glutamate. GABA neurons can convert the absorbed glutamine into glutamate, and then into GABA. Subsequently, GABA is excreted and acts on the next neuron. Upon the binding of GABA to the GABA_A_ receptor, it prompts the opening of chloride ion channels within the receptor, thereby giving rise to either the inward or outward flow of chloride ions. The predominant direction of movement is the inward flow. **(B)** GABA_B_ receptors’ workflow. GABA_B_ receptors are capable of anchoring to Ca^2+^ and K^+^ channels on the cell membrane. Through the dynamic modulation of the closure of these ion channels, it restricts the influx of Ca^2+^ and augments the influx of K^+^, thereby attaining an inhibitory hyperpolarized state of the membrane potential.

In bacteria, GABA is primarily involved in the survival ability in an acidic environment. *Escherichia coli* and other enterobacteria utilize the H^+^ consuming reaction catalyzed by gad to reduce the concentration of H^+^ around the microorganism itself, and survive in the stomach acidity before reaching the intestine (Figure 1B) (Sarasa et al., 2020). In the vertebrates, GABA primarily acts on GABA receptors, triggering ion exchange across the neuronal membrane, altering current signals, and generating inhibitory potentials, thereby inhibiting neuronal excitation (Taylor et al., 2003). Particularly, GABA can act on ionotropic receptors such as GABA_A_ and GABA_C_ receptors, as well as the metabotropic receptor GABA_B_ receptor in the brain (Figure 2) (Felice et al., 2022).

GABA_A_ receptors are pentameric ligand-gated ion channels widely expressed throughout the central and peripheral nervous system (Luo and Balle, 2022). When GABA binds to the GABA_A_ receptor, it causes the chloride ion channels on the receptor to open, and leads to either the influx or efflux of chloride ions, with the predominant movement being the influx (Figure 2A) (Kim and Hibbs, 2021). The opening of the chloride ion channels by GABA helps stabilize the resting potential of cells during the activation of excitatory receptors and makes it more difficult for neurons to generate excitatory action potentials and release neurotransmitters (Bormann, 1988; Kim and Hibbs, 2021).

GABA_B_ receptors are G-protein coupled receptors and primarily exist in a heterodimeric form. Different from the GABA_A_ ion channels, GABA_B_ receptors can anchor to Ca^2+^ and K^+^ channels on the cell membrane (Figure 2B) (Bormann, 1988). By dynamically regulating the closure of these ion channels, it inhibits the influx of Ca^2+^ and promotes the influx of K^+^ to achieve an inhibitory hyperpolarized state of the membrane potential. Additionally, it can dynamically regulate the switches of the G-proteins it anchors to, realizing the activation of guanine nucleotide-binding proteins and indirectly producing the second messenger cAMP to exert its effects (Bettler et al., 2004).

The GABA_C_ receptor is a subclass of the GABA_A_ receptor, and is often referred to as the GABA_A_ rho receptor with three different subtypes ρ1-3 (Olsen and Sieghart, 2008). Its structure and function are largely similar to those of GABA_A_ receptors. It is categorized separately due to being insensitive to both bicuculline and baclofen (Johnston, 2013).

In the brain, GABA primarily plays a role in intermediate neurons, which are notably rich in the enzyme GAD. Experimental findings have revealed that the removal of the GAD enzyme from the mouse brain tissue results in a significant reduction in detectable GABA levels (Ji et al., 1999).

In the synaptic cleft, any excess GABA that remains unabsorbed after transmission is re-uptaken by astrocytes, where it is converted into glutamine (Figure 2A). This glutamine is then utilized by glutamatergic neurons to synthesize glutamate. Subsequently, the released glutamate is taken up by GABAergic neurons to regenerate GABA. Through the coordinated activity of glutamatergic neurons, GABAergic neurons and astrocytes, GABA stays at a balanced level, and the neural network maintains a state of equilibrium, which is essential for the proper functioning of the brain (Mazzoli and Pessione, 2016; Steel et al., 2020).

In patients with anxiety and insomnia, signs of excessive consumption of GABA were detected. This may be related to the overactivity of GABA transaminase, which causes excessive conversion of GABA into succinic semialdehyde (SSA), reducing the neuroinhibitory effect of GABA (Liu M. Q. et al., 2022). Adding some GABA transaminase inhibitors can be effective in the treatment of anxiety and insomnia (Di Pierro et al., 2024; Liu M. Q. et al., 2022; Srichomphu et al., 2022).

## 5 Therapeutic effects of gut derived GABA on anxiety and insomnia

### 5.1 Anxiety

Gut derived GABA encompasses GABA obtained from dietary sources as well as GABA produced by the resident gut microbiota, and represents a significant source of GABA in the human body (Duranti et al., 2020; Liwinski et al., 2023). Direct oral GABA supplements and dietary food, for instance potatoes, tomatoes, rice, tea and beans, are primary sources of gut derived GABA (Tables 1, 2) (Cai et al., 2014; Hinton and Johnston, 2020; Kim et al., 2015; Lei et al., 2023; Liwinski et al., 2023; Vann et al., 2020; Yan et al., 2021). Moreover, bacteria can convert glutamate into GABA through a process that utilizes protons (Feehily and Karatzas, 2013). Probiotic bacteria such as *Bifidobacterium*, *Lactobacillus* and *Lactiplantibacillus* can produce GABA and significantly increase the GABA level in the intestines (Table 3) (Barrett et al., 2012; Duranti et al., 2020; Luck et al., 2021; Watanabe et al., 2022). Notably, studies have shown that gut derived GABA can treat anxiety and insomnia by exerting a widespread influence on hormone levels, secretion of signaling molecules, gene expression, and various other physiological changes (Abdou et al., 2006; Liwinski et al., 2023; Yamatsu et al., 2015).

**Table 1:** Gut derived GABA exhibits therapeutic effects in the treatment of anxiety.

Subject	Dosing	Frequency	Function	References
Human	100 mg	3 administrations, separated by 7-day intervals	Anxiety ↓, Brain alpha wave ↑, beta wave ↓, saliva IgA level ↑	Abdou et al. (2006)
Human	100 mg	Everyday for 8 weeks	Stress ↓, Alpha & beta band brain waves ↓	Yoto et al. (2012)
Human	0–50 mg (beverage)	Once a time	Psychological fatigue ↓, Salivary chromogranin A & cortisol levels ↓	Kanehira et al. (2011)
Human	28 mg GABA (chocolate)	Once a time	Psychological stress ↓, HRV (heart rate variability) ↑	Nakamura et al. (2009)
Human	Vegetable tablets containing GABA	Once a time	Sympathetic nervous activity ↓, blood pressure ↑	Okita et al. (2009)
Human	GABA-fortified oolong tea	Once a time	Chronic stress ↓, autonomic imbalance impact ↓, HRV ↑	Hinton et al. (2019)

**Table 2:** Gut derived GABA exhibits therapeutic effects in the treatment of insomnia.

Subject	Dosing	Dosing frequency	Function	References
Human	300 mg	Everyday for 4 weeks	Sleep latency ↓, sleep efficacy ↑	Byun et al. (2018)
Human	100 mg	30 min before going to bed, everyday	Sleep latency ↓, NREM sleep ↑	Yamatsu et al. (2015)
Human	100 mg	Every day for a week	Plasma GABA levels ↑, sleep latency ↓, total non-REM sleep duration ↑	Yamatsu et al. (2016)
Human	GABA tea	Everyday for a month	Sleep onset latency ↓, REM onset latency ↑	Cheng and Tsai (2009)

**Table 3:** Summary of bacterial strains producing GABA.

Subject	Strain	Function	References
Human	*Bifidobacterium breve M-16V*	Mood and sleep scores ↑	Mutoh et al. (2024)
Human	*Lactobacillus brevis DPC6108*	Gut GABA capacity ↑	Barrett et al. (2012)
Mice	*Bifidobacterium adolescentis IM38*	Anti-anxiolytic-like effect ↑, Corticosterone and IL-6 blood levels ↓, Blood TNF-α level ↓	Jang et al. (2018)
Mice	*Bifidobacterium longum DD98*	Depression and anxiety-like behaviors ↓, Serotonin, GABA, NPY, BDNF expression ↑	Jin et al. (2023)
Mice	*Lactobacillus rhamnosus (JB-1)*	Anxiety and depression-related behaviors ↓, GABA_A,B_ receptors sub-types in specific brain regions ↑	Janik et al. (2016)
Mice	*Lactobacillus plantarum 286 and 81*	Anti-depressant-like and anxiolytic-like effects ↑	Barros-Santos et al. (2020)
Mice	*Lactobacillus plantarum HJZW08*	Anxiety-like and depressive-like behaviors ↓, neuroactive molecules levels ↑	Wu et al. (2022)
Mice	*Lactobacillus rhamnosus GG*	Anxiety behaviors ↓, BDNF and GABA receptors levels in hippocampus and amygdala ↑	Zhou B. et al. (2022)
Mice	*Lactiplantibacillus plantarum LZU-J-TSL6*	Anxiety disorder ↓, hippocampal region GABA content ↑, some anxiety-related markers ↑	Liu G. et al. (2022)
Mice	*Lacticaseibacillus rhamnosus GG*	Anxiety and depressive-like phenotype ↓	Faucher et al. (2022)
Monkey	*Bifid Triple Viable Capsules* (*Bifidobacterium*, *Lactobacillus*, and *Enterococcus faecalis*.)	Plasma GABA levels ↑, stress responses and gut microbiota dysbiosis ↓	Zhao et al. (2022)

Studies indicate that both the direct administration of 100 mg of GABA and the consumption of GABA-containing foods exhibit a certain therapeutic effect on anxiety-related behaviors (Table 1) (Barros-Santos et al., 2020; Faucher et al., 2022; Yoto et al., 2012). The administration of the GABA-producing strain *Bifidobacterium adolescentis IM38* significantly improved anxiety-like behaviors in mice, and this effect was blocked by flumazenil, a benzodiazepine receptor antagonist (Jang et al., 2018). This finding suggests that gut derived GABA exerts their influence through GABA-regulated sites, potentially acting on GABA receptors in the brain. Moreover, administering probiotics *Lactiplantibacillus plantarum SNK12*, which produces GABA, has been shown to alleviate anxiety and reduce salivary cortisol levels in humans (Watanabe et al., 2022). Similar phenomena are also observed in animal models (Cai et al., 2022).

### 5.2 Insomnia

Clinical trials have provided evidence that the administration of 100 mg of GABA significantly improves sleep quality, which is reflected by a reduction in sleep latency and an increase in the duration of non-REM sleep (Table 2) (Yamatsu et al., 2015; Yamatsu et al., 2016). Similar positive effects on sleep quality have been observed in clinical trials involving the consumption of GABA tea (Cheng and Tsai, 2009). In animal studies, a significant decrease in peripheral blood GABA concentration of sleep-deprived monkeys, accompanied by a substantial increase in peripheral blood NE and cortisol, both indicative of HPA axis upregulation (Zhao et al., 2022). Supplementation with probiotics producing GABA significantly reduced the levels of hormones associated with the HPA axis and increased peripheral blood GABA levels, suggesting a therapeutic effect. Similar findings were also reported in mouse experiments. Mice fed GABA black tea exhibited a significant decrease in sleep latency period induced by pentobarbital sodium, accompanied by a substantial increase in the duration of effective sleep (Zhao et al., 2015). Administration of 100 mg/kg GABA not only significantly reduced sleep latency and increased sleep duration, but also elevated the expression levels of GABA receptor mRNA in the mouse brain, proving that supplementing with gut derived GABA can indeed enhance GABAergic signaling in the brain and improve sleep (Chen et al., 2021; Kim et al., 2019; Liu D. et al., 2022).

### 5.3 Dosage and side effects

Studies indicated that a direct administration of 100 mg of GABA represents an appropriate dosage for the treatment of anxiety and insomnia. This dosage can exert positive effects in terms of alleviating anxiety, shortening sleep latency, and enhancing sleep quality (Abdou et al., 2006; Yamatsu et al., 2015; Yamatsu et al., 2016; Yoto et al., 2012). Research has demonstrated that even when a relatively high dose, such as 6 g of GABA per day, is administered, no obvious side effects are observed in volunteers, suggesting the favorable biosafety of GABA (Li et al., 2015). However, it is noteworthy that current studies have revealed that GABA is not conducive to the anti-tumor response. Multiple investigations have elucidated that GABA can inhibit dendritic cell mediated T-cell recruitment and activation (Huang et al., 2022; Zhang et al., 2021). Therefore, it is not advisable to use gut derived GABA as a drug treatment for cancer patients.

### 5.4 Probiotics and engineered bacteria

It is worth noting that in addition to the dietary and orally administered GABA presented in Tables 1, 2, the utilization of GABA-producing bacteria has emerged as an innovative therapeutic strategy for anxiety and insomnia (Table 3). A substantial body of research demonstrates that GABA-producing bacteria can effectively mitigate these disorders. These bacteria exert diverse effects on the body, such as diminishing stress factor levels, augmenting GABA concentrations, and modulating the levels of GABA receptors and their corresponding mRNAs in the brain (Janik et al., 2016; Jin et al., 2023; Liu G. et al., 2022; Zhou B. et al., 2022).

Unlike orally ingested GABA, which is rapidly eliminated from the body, GABA-producing gut bacteria can colonize the host’s gastrointestinal tract for an extended period. This persistent colonization enables continuous GABA synthesis, thereby conferring prolonged therapeutic benefits (Pokusaeva et al., 2017). Additionally, the impact of these bacteria on the gut’s ecological environment is persistent over time. Significantly, the employment of probiotic strains has the significant advantage of imposing a lower metabolic burden on organs such as the liver compared to traditional small molecule drugs (Li et al., 2015; Oketch-Rabah et al., 2021).

Moreover, through microbiome sequencing, it has been found that some bacteria can either promote or inhibit the growth of GABA-producing bacteria. Supplementation with *Limosilactobacillus fermentum L18* can significantly increase the abundance of *Bifidobacterium* and *Lactobacillus* (Kaur et al., 2023). This might be due to that these strains can optimize the acidic environment for GABA production by regulating the intestinal pH value and metabolite exchange. However, some strains, such as *Enterobacteriaceae*, can reduce the abundance of GABA-producing bacteria. This could be due to the production of substances like lipopolysaccharides by these strains, which hinder the growth of GABA-producing bacteria (Zeng et al., 2024). Thus, the supplementation of GABA-producing bacteria is still an effective method. In the future, the administration of combined bacterial agents could be considered (Zhao et al., 2022).

Apart from certain natural probiotic strains like *Bifidobacterium* and *Lactobacillus*, genetically engineered bacterial strains have also been harnessed in the treatment of anxiety. To date, numerous studies have endeavored to employ engineered probiotics to express GABA and address anxiety (Lebovich and Andrews, 2022, 2023; Pan et al., 2022; Pokusaeva et al., 2017). The advantage of using engineered bacteria for anxiety treatment resides in the precise regulation of GABA production, for instance treating anxious mice using an engineered *Lactococcus lactis* strain (Pan et al., 2022).

Moreover, the majority of these studies have adopted *Escherichia coli Nissle 1917* (*EcN*) (Lebovich and Andrews, 2022, 2023). *EcN* is a renowned probiotic strain of *Escherichia coli*. Owing to its probiotic properties and well-defined manipulable genetic background, it is currently extensively utilized as a vector in engineered bacteria therapy (Lynch et al., 2022). When engineered *EcN* is employed to produce GABA, the GABA yield can attain as high as 17.9 g/L (Lan et al., 2021). Another benefit of engineered bacteria is their proficiency in GABA production, as they can overexpress gadB and gadC proteins within the bacteria. Nevertheless, at present, research articles on the application of *EcN* that produces GABA to treat neurological diseases remain relatively scarce, presenting an area worthy of future exploration.

There are several issues regarding probiotics that need to be addressed. The introduction of gut microbiota can alter intestinal metabolic activities in the host, which may have adverse effects on a very small number of individuals. For example, the probiotics *L. acidophilus* and *Bifidobacterium* can convert primary bile salts into secondary bile salts (Ruseler-van Embden et al., 1995). Most secondary bile salts are reabsorbed to enter the liver for metabolism. If the liver function is abnormal and unable to metabolize secondary bile salts properly, it may lead to their accumulation in the liver, exerting toxic effects on liver cells and further aggravating liver damage (Zawistowska-Rojek and Tyski, 2018). Moreover, some probiotics may carry antibiotic-resistance genes, which poses a risk of potential transfer of these genes within the gut microbiota (Merenstein et al., 2023). These issues need to be considered and addressed in the future studies.

### 5.5 Gut microbiota

**Figure 3:**
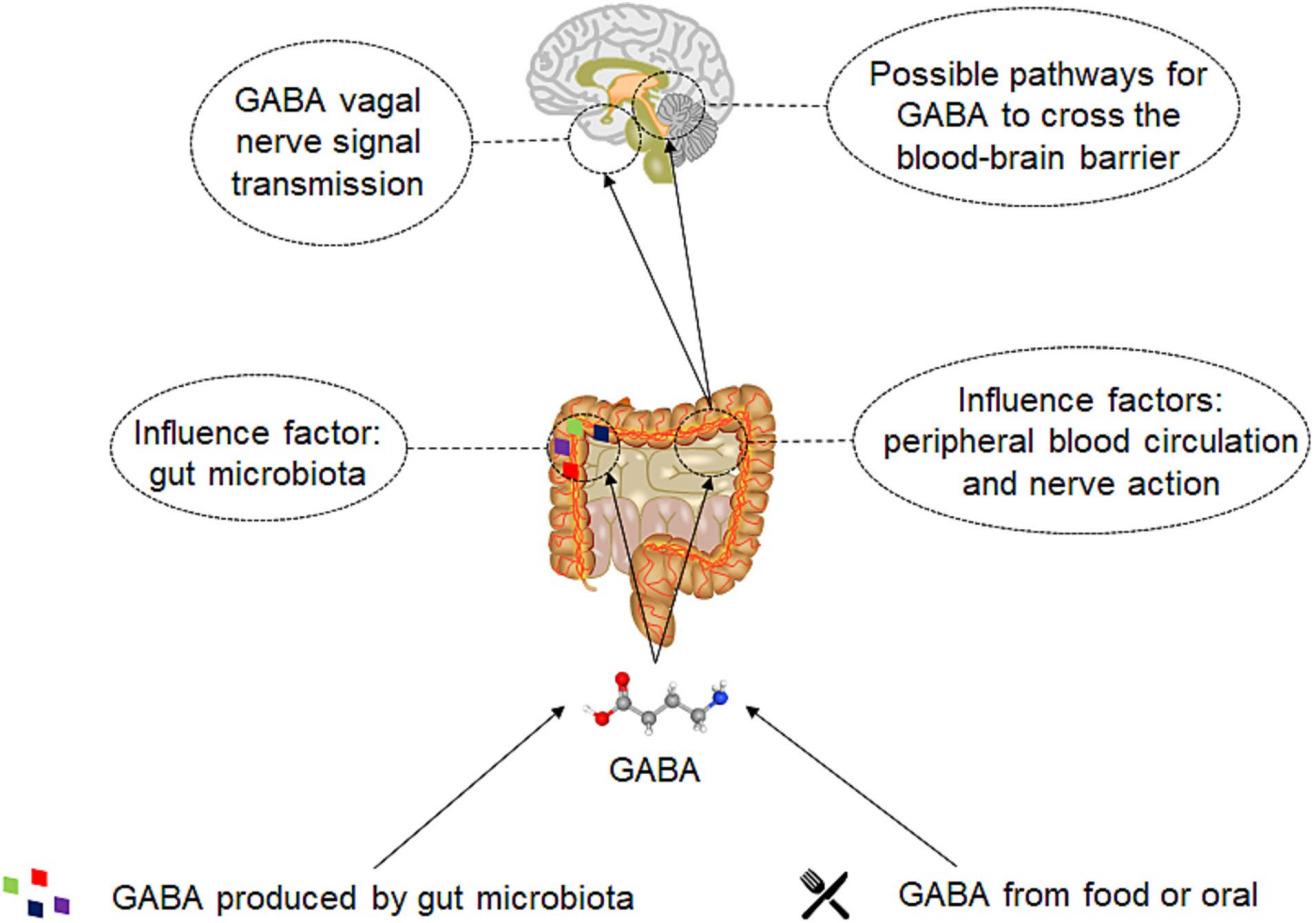
The mechanism of gut derived GABA on human body. Two sources gut derived GABA: one is obtained through direct oral intake or via food, and the other is generated by the gut microbiota. Subsequently, GABA will directly act on the gut microbiota and have a direct impact on its metabolism. A significant portion will also be absorbed by the gut, enter the bloodstream, or act on enteric neurons and the vagus nerve. GABA that acts on the enteric nerves and vagus nerve can be transmitted through neural signals and affect the brain. GABA that enters the bloodstream may eventually interact with the brain by crossing the blood–brain barrier.

To optimize the application of gut derived GABA, it is crucial to summarize and investigate the underlying mechanisms of its effects. When GABA is taken orally and enters the intestinal tract, it will first be affected by the body’s intestinal microbiota (Figure 3). GABA is reported to act as a critical growth factor for certain strains of intestinal bacteria in the human gut microbiota, leading to an increase of these specific microbial communities within the host (Strandwitz et al., 2019). GABA also serves as a carbon and nitrogen source for the gut microbiota. Within bacteria, GABA can be further converted into succinic semialdehyde (SSA) by GABA transaminase. Subsequently, SSA is transformed into succinate by the enzyme succinic semialdehyde dehydrogenase, which then enters the tricarboxylic acid (TCA) cycle for further metabolic processing (Sarasa et al., 2020).

### 5.6 GABA absorption

#### 5.6.1 Small intestine epithelial cell transport: SLC family

**Figure 4:**
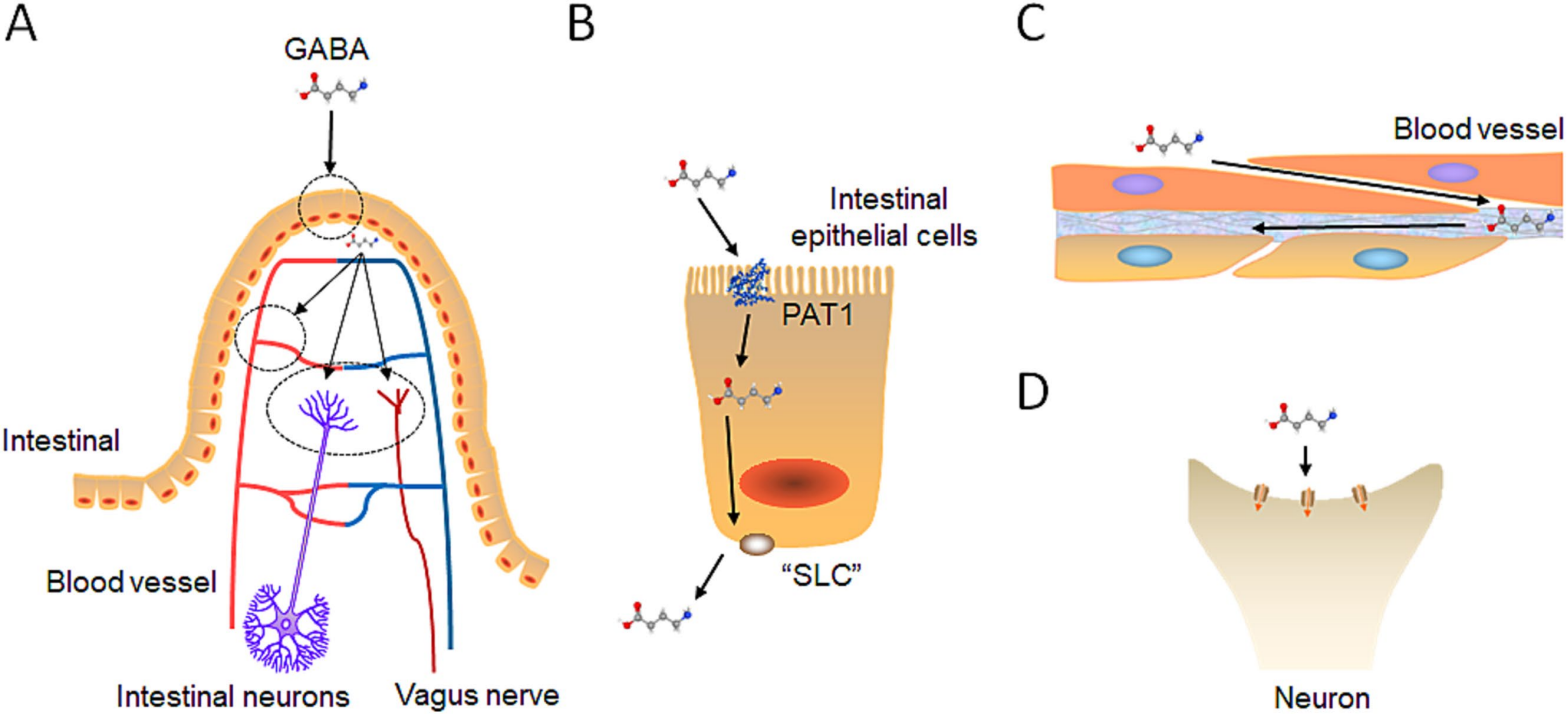
Exogenous GABA intestinal absorption pathway. **(A)** GABA intestinal absorption diagram: Initially, GABA is taken up by intestinal epithelial cells. Then, it diverges into three pathways: bloodstream entry, interaction with enteric neurons, and connection with the vagus nerve. **(B)** GABA intestinal transport: GABA crosses the small intestine membrane via the PAT protein on the basolateral side and exits through a probable SLC family protein on the apical side. **(C)** GABA integrates into the blood circulation: GABA penetrates the fenestrated capillaries within the intestinal lining to join the bloodstream. **(D)** GABA binds to receptors: GABA interacts with GABA receptors on intestinal neurons and the vagus nerve, as previously described, to exert its effects.

The initial site of interaction between gut derived GABA and the human body is the intestinal tract. In the intestine, gut derived GABA is absorbed by intestine epithelial cells (Figure 4). The intestine normally employs passive absorption to transport small-molecule substances, such as amino acids (Nácher et al., 1994; Thwaites et al., 2000). GABA transporters in the intestines of both rodents and humans facilitate both active and passive absorption of GABA (Nácher et al., 1994; Thwaites et al., 2000). Proton-coupled amino acid transporter 1 (PAT1) has been confirmed as the GABA transporter responsible for transporting GABA across the apical membrane of intestinal epithelial cells into the cells (Figure 4B) (Chen et al., 2003).

It is speculated that small intestinal epithelial cells exclude GABA from the basal membrane into the extracellular space of the submucosal layer through one of the solute carrier transporters (SLC) family proteins (Figure 4B). SLC transporters comprise approximately 350 members belonging to 55 families, with some SLC proteins exhibit bidirectional transport functions (Kristensen et al., 2011). For example, SLC6A1 (GAT1) mediates the transport of GABA together with sodium and chloride ions, and is responsible for the reuptake of GABA from the synapse. The direction and magnitude of GABA transport are determined by thermodynamic conditions, influenced by factors such as membrane potential and the concentrations of Na^+^, Cl^−^, and GABA (Mattison et al., 2018). SLC family proteins with bidirectional transport mechanisms or one that transports GABA from intracellular to extracellular spaces may exist at the basal membrane of small intestinal epithelial cells, facilitating the transport of GABA (Zafar and Jabeen, 2018). Further research is necessary to confirm this possibility.

#### 5.6.2 Capillary absorption

When GABA is transported by small intestinal epithelial cells to the lamina propria, it partially enters the capillaries to participate in blood circulation (Figure 4C). The capillaries in the lamina propria are fenestrated, called “fenestrated capillary,” meaning that they have intracellular pores or “windows” with a diaphragm that penetrate the endothelial lining (Augustin and Koh, 2017). These pores facilitate the exchange of water and allow the passage of solutes, such as small peptides, between plasma and interstitial fluid. Fenestrated capillaries are found throughout various tissues, including the choroid plexus of the brain, certain endocrine organs, kidney filtration sites, and intestinal absorptive regions (Augustin and Koh, 2017). Given that GABA is significantly smaller than peptides, it can theoretically pass through the pores of fenestrated capillaries. Consequently, research has demonstrated that GABA derived from the gut can effectively elevate GABA levels in the bloodstream of both humans and animal models (Yamatsu et al., 2016; Zhao et al., 2022).

#### 5.6.3 Vagus nerve absorption

GABA is transported by small intestinal epithelial cells to the lamina propria, where some enter the bloodstream, and some act on the intestinal nerve plexus, which causes intestinal muscles and glands to respond and transmit signals to other parts such as the brain through the vagus nerve (Figure 4D). Interestingly, administering *Lactobacillus rhamnosus (JB-1)*, a probiotic strain producing GABA, has been shown to improve anxiety-and depression-related behaviors in mice (Bravo et al., 2011). However, after surgically removing the vagus nerves in mice fed with the probiotic, the behavioral improvements were lost, indicating that the vagus nerve serves as a major modulatory constitutive communication pathway between gut-exposed bacteria and the brain.

#### 5.6.4 Hypothalamus—hormone secretion

After receiving the signals of gut derived GABA, the afferent fibers of the vagus nerve in the gastrointestinal tract transmit the messages through neuronal synapses, converting them into cholinergic signals (Qu et al., 2024). These signals are then transmitted to the nucleus tractus solitarius (NTS) in the medulla oblongata. Subsequently, the NTS communicates with the locus coeruleus, which sends signals onward to either the amygdala or the thalamus (Breit et al., 2018). Additionally, the hypothalamus directly receives signals from GABA in the peripheral blood. Structurally, it is closely connected to the primary capillary plexus within the pituitary portal system (Ilgin, 2020). Studies have shown that injecting competitive antagonists of GABA_A_ receptors into the PVN significantly increases stress-induced cortisol secretion, whereas selective GABA receptor antagonists notably reduce it (Cullinan et al., 2008). Moreover, supplementation with GABA-producing probiotics inhibits the activated HPA axis. Indeed, this indirectly provides evidence that GABA in peripheral blood can act on the hypothalamus to regulate the HPA axis (Cai et al., 2022; Watanabe et al., 2022).

### 5.7 Brain

#### 5.7.1 The pathway of gut derived GABA entering the brain

The impact of gut derived GABA on the brain has been a contentious topic, with a central question being the permeability of the blood–brain barrier (BBB). While some studies suggest that GABA cannot penetrate the BBB in mammals, other research indicates that GABA can traverse it, albeit in modest quantities (Boonstra et al., 2015; Frey and Löscher, 1980; Knudsen et al., 1988; Toth and Lajtha, 1981; Van Gelder and Elliott, 1958). Analyzing the structural aspects of the BBB could provide a theoretical basis to support the potential passage of GABA through this barrier.

The BBB consists of adjacent capillary endothelial cells interconnected by impermeable tight junctions, requiring molecules to enter through active uptake by specialized transporter proteins or by diffusing into the BBB cells (Abbott et al., 2010; Brightman and Reese, 1969; Pardridge, 2005, 2007). Among these transporters, a crucial group is the SLC6 family, one of the largest SLC families comprising 20 genes that encode highly similar transporter proteins. The SLC6 family, abundantly expressed in the brain, includes GABA transporters such as GAT1 (SLC6A1), GAT2 (SLC6A13), GAT3 (SLC6A11), and BGT1 (SLC6A12). These transporters are responsible for the transport of neurotransmitters into and out of the brain, thereby regulating neurotransmitter homeostasis (Abbott et al., 2010). Studies have shown that GAT2/BGT-1 expressed in epithelial cells serve as GABA transporters at the BBB to transport GABA (Takanaga et al., 2001). In addition, GAT2/BGT-1 has been found to be widely expressed in the mouse brain. Notably, GAT2 is expressed on both the apical and basal membranes of epithelial cells, exhibiting bidirectional transport capability. In contrast, BGT1 is expressed solely on the basal membrane. GAT2 at the BBB facilitates the transport of GABA.

Extensive studies have observed increased concentrations of brain GABA and mRNA expression levels of GABA receptors following administration of gut derived GABA (Jin et al., 2023; Watanabe et al., 2022; Zhou H. et al., 2022). These findings collectively support that gut derived GABA can modulate the brain’s GABAergic system, potentially alleviating neurological disorders by enhancing GABAergic signaling in the brain.

## 6 Future direction

Currently, a large portion of the global population is suffering from anxiety and insomnia, grappling with the challenges posed by these mental health disorders. The current sedatives and sleep aids available for treating anxiety and insomnia often come with side effects like physical dependence and withdrawal symptoms (Shyken et al., 2019). Hence, the development of novel interventions that address anxiety and insomnia without such drawbacks remains necessary.

GABA, as a natural neurotransmitter in the human body, poses minimal harm even in high doses (Li et al., 2015). The supplementation of GABA through gut derived sources holds great promise. Oral GABA, GABA-enriched diets, and probiotic strains producing GABA have demonstrated positive and effective outcomes in treating anxiety and insomnia. However, oral GABA has several limitations, which include: First, low blood–brain barrier permeability: Unlike benzodiazepines, GABA does not efficiently cross the blood–brain barrier, limiting its ability to exert strong pharmacological effects. Overall, the efficiency of GABA crossing the blood–brain barrier is not very high. Second, low bioavailability: The bioavailability of GABA when ingested is relatively low, and it is quickly eliminated from the body, which makes it challenging to sustain therapeutic efficacy. Third, non-specific effects: GABA is widely distributed throughout the body, not only in the central nervous system but also in many other tissues and organs. This wide distribution may lead to non-specific effects rather than targeting specific diseases or symptoms. An increasing amount of research has found that GABA functions as a signal molecule in the immune system. Several studies have clarified that GABA is not beneficial for the anti-tumor response. So, it is not recommended to provide gut derived GABA as a drug treatment for cancer patients.

Probiotic and engineered bacteria capable of producing GABA present promising avenues for the treatment of anxiety and insomnia. These bacteria can colonize in the human gut and release GABA promptly. There remain several aspects that warrant further exploration. For instance, determining the optimal drug dosage of probiotics for human treatment and, notably, laying particular emphasis on considering the safety of engineered foreign genes. Future research efforts should be intensified to not only optimize the drug dosage regimens but also to conduct rigorous and systematic investigations into the safety of foreign genes. Only through such meticulous exploration can we hope to translate these promising approaches into reliable and effective therapeutic solutions, thereby offering a new ray of hope for the countless individuals suffering from anxiety and insomnia, and revolutionizing the landscape of mental health treatment.
